# Exploring Attitudes to Conception in Partners and Young Women with Gynecologic Cancers Treated by Fertility Sparing Surgery

**DOI:** 10.31557/APJCP.2020.21.9.2609

**Published:** 2020-09

**Authors:** Prue Standen, Paul A Cohen, Yee Leung, Ganendra Raj Mohan, Stuart G Salfinger, Jason Tan, Caroline Bulsara

**Affiliations:** 1 *Joan Kirner Hospital, St Albans, Victoria, Australia. *; 2 *King Edward Memorial Hospital, Subiaco, Western Australia, Australia. *; 3 *St John of God Hospital, Subiaco, Western Australia, Australia. *; 4 *Division of Obstetrics and Gynaecology, Faculty of Medical and Health Sciences, University of Western Australia, Crawley, Western Australia, Australia. *; 5 *Institute for Health Research, University of Notre Dame Australia, Fremantle, Western Australia, Australia.*; 6 *School of Medicine, University of Notre Dame Australia, Fremantle, Western Australia, Australia. *; 7 *WOMEN Centre, West Leederville, Western Australia, Australia. *

**Keywords:** Gynecologic cancer, fertility preservation, conception; partners

## Abstract

**Background:**

Approximately 20% of women with gynecologic cancers are under age 40 and with delayed childbearing, women may be diagnosed before their first pregnancy. Although fertility preservation is a priority for many women, attitudes to conception have not previously been investigated in these patients or their partners. We explored attitudes to conception in partners and young women, following fertility preserving treatment for gynecologic cancers.

**Methods:**

A total of 16 telephone interviews were conducted with a purposive sample of patients who had had an early stage gynecologic cancer or borderline ovarian tumor treated by fertility sparing surgery in Western Australia between January 1st, 2005 to December 31st, 2016. The interviews were audio recorded, transcribed and thematic analysis was conducted.

**Results:**

Four main themes were identified: (i) Emotions at diagnosis and perception of information given; (ii) Discussions of fertility and factors affecting childbearing; (iii) Role of partners in decision making and relationship pressures; (iv) Decision for treatment and postoperative regrets.

**Conclusions:**

Regret and relationship breakdown were commonly reported. Women need appropriate support including inviting their partners to attend clinic appointments and may need several appointments before treatment. Regret was commonly reported by women who opted for completion surgery.

## Introduction

In 2018 there were an estimated 8.6 million new cancer diagnoses and 4.2 million deaths worldwide (Bray, 2018). Of these cases more than 1.3 million were malignancies of the female genital tract. Up to 21% of women with gynecologic cancers are under 40 years of age at the time of diagnosis (Feichtinger and Rodriguez-Wallberg, 2016; Lagana et al., 2017; Makar and Trope, 2001). A high proportion will have early stage and potentially curable disease, and thus fertility preservation is a priority for many patients. With global trends to delayed childbearing, many women may be diagnosed before their first pregnancy. A diagnosis of cancer has been recognized to be a stressful experience causing a significant impact on a social, sexual, and psychological function and wellbeing amongst women (Lagana et al., 2017). The psychological distress related to infertility is recognized as most significant in those who have not yet had children and desire to do so (Lagana et al.). 

Decisions around fertility preservation are personal and multifactorial (Zapardiel et al., 2016) and few studies have explored patients’ experiences and decision-making. Fertility preserving surgical treatment may be offered for appropriately selected patients with stage IA1 to IB1 cervical cancer, stage IA grade 1 endometrial cancer and borderline ovarian tumors (Feichtinger and Rodriguez-Wallberg, 2016). Ovarian cancer requires a slightly different approach and fertility preservation can be considered for patients with germ cell and sex cord tumors, and stages IA and IC1, grade 1-2 epithelial carcinomas after appropriate counseling. 

A study by Carter et al., (2010) noted that thirty percent of women who had undergone radical trachelectomy were not sexually active at 6 months post-surgery due to being “*somewhat… to very afraid*” to have intercourse and furthermore that up to 50% of women with early stage cervical cancer who elect fertility preservation do not subsequently attempt conception. Infertility following cancer treatment is a recognized concern with subsequent effects on perceived quality of life. Potential barriers include lack of adequate counseling and information, pre-existing or subsequent infertility, cancer-related complications, sexual dysfunction, psychological issues, socio-economic background as well as religious and spiritual beliefs. We aimed to explore attitudes to conception in partners and young women with gynecologic cancers treated by fertility sparing surgery with the intention of exploring the factors that influence decisions regarding childbearing for these patients. 

## Materials and Methods 

*Recruitment*

Participants were selected by purposive sampling from the Tumor Board database of the Western Australian Gynecologic Cancer Service. They were eligible for inclusion if they had an early stage gynecologic cancer treated by fertility sparing surgery in Western Australia from January 1^st^, 2005 to December 31^st^, 2016 and were fluent in English. Patients meeting the inclusion criteria were invited to participate via mail. An opt-out method was used and patients received a follow-up telephone call inviting them to participate. Participants provided written informed consent and gave permission for the interviews to be recorded. Ethics approval was granted by the governing committees of both the private and public hospital sites where patient recruitment was conducted (RGS0000000540 and reference #1215).

*Interviews*

Semi-structured interviews lasting approximately 1 hour were conducted over the telephone by the principal investigator. There were no non-participants present. The interview guiding questions centred around the participants recollections of the time of diagnosis, options for fertility sparing surgery and fertility services offered, as well as their perception of what effect this diagnosis may have had on their relationships and sense of future planning. Partners were asked similar and corresponding questions about their recollections of the time of diagnosis and perceptions of how their partner was treated, as well as whether they felt involved and respected by the medical staff. There were no repeat interviews. Field notes were made during each interview and the interviews were recorded and transcribed verbatim and there were no participants who requested a copy of their transcript.

*Analysis*

Inductive thematic analysis as described by Braun (2006) (Braun, 2006) was conducted using the software NVIVO 12 (QSR international 2018) for data analysis management. An inductive approach meant that the themes identified were strongly linked to the data (Patton, 1990). A second researcher read and coded the transcripts independently and consensus into emerging themes was reached. 

## Results

Overall, 49 patients were eligible for inclusion and were invited to participate in this study. Of these, 3 patients declined whilst there were 32 non-responders. Subsequently, 14 patients and 2 partners participated in the study and participant characteristics are shown in Table 1. 

Data analysis identified 4 key themes. These were (i) Emotions at diagnosis and perception of information given; (ii) Discussions of fertility and factors affecting childbearing; (iii) Role of partners in decision making and relationship pressures; (iv) Decision for treatment and postoperative regrets. Key findings are highlighted in Table 2 and will be subsequently discussed in detail in the results.

*Emotions at diagnosis of early stage gynecologic cancer and perception of information given*

The overwhelming emotional response in young women diagnosed with early stage gynecologic cancer was of a feeling of shock and uncertainty of the future. Kim said,

 “*I was highly emotional at the time and I had different people coming in with me, the support people. Being diagnosed was an emotional rollercoaster for me.*”

And Laura explained, 

 “*Going to the oncology clinic, that threw me. ‘How come I’m here, I don’t have cancer.’.*” 

Another participant (Hannah) commented on how frightening the experience was for someone to be given the diagnosis of cancer. 

Some women found it confronting to be given the diagnosis and then to suddenly consider their plans for the future, including childbearing. Nonetheless there was the contrasting sense of relief and hope that having a diagnosis would allow for the appropriate treatment and the knowledge that for some people, the diagnosis was worse.

Hannah said: “*sitting in the waiting room, waiting to go for a biopsy and you’ve got other people sitting there that have stage 4- that could have been me in a few years. It’s very confronting.*”

Following the diagnosis, women said that they wanted more information relating to prognosis but also the opportunity to make a decision without feeling pressured. Importantly, for the women who said that they didn’t feel supported there was a temporal association with another significant event such as an emergency procedure or a stressful birth experience resulting in a sick newborn child. One participant spoke of the lack of options presented to her and lack of explanation or advice from the clinician. Alice explained,

 “*I know that it was all emergency surgery, I don’t feel that at the time that there was enough explanation or enough discussion about it. They made me feel like I had no choice in the matter. It wasn’t a good experience, I remember getting a stern talking to by the doctor. What options are there in this scenario. I felt that I had no other option than a hysterectomy otherwise I would die*.”

One of the partners, Ben, explained:

“*I didn’t really look for additional support. I just wanted her to be looked after. A lot of the time I had to fish for information. I had to ask questions when maybe we should have just been told.*”

*Discussions of fertility and factors affecting childbearing*

Our findings showed that half of the women interviewed had not thought about their future fertility or timing of conception prior to their diagnosis. One participant (Anna) commented, 

 “*it got me thinking about the possibility of not having children in the future and the idea that the clock is ticking. I hadn’t really considered losing that option. It didn’t make me go ‘I want to freeze my eggs’.*”

Another (Sophie) spoke of ‘*keeping options open*’, “*I didn’t think that I wanted children, I always thought that I would adopt but you always want to keep your options open. If we were to have children, my partner is more interested in carrying the child’’.*

And yet another (Kim) also highlighted the perceived lack of options presented to her and that she was not at a juncture in her life which encouraged her to think about having children:* “One doctor spoke with me about fertility, they talked about the risks. It wasn’t really thoroughly discussed but I’m sure that someone must have asked me what my plans were. I didn’t have any because I was single. I can’t think of any options that were given to me in terms of family planning. I didn’t feel that any information was lacking, I just let life do what it needs to do. I don’t plan on having a family but if it happens, it happens. If I had had to have a hysterectomy, it would have changed the way that I thought about my fertility.*” The responses from participants who had not considered children or were not in relationships and considering children were in contrast to those women who already had children or who had already thought about having children in the future. Several of these women proceeded to IVF as can be demonstrated below. 

Claire: “*I want more children, this was all discussed with the care team.*”

Julie: “*We knew that we wanted to have kids right from the start. We just knew that it was a possibility that we would have to do a lot of hard work and that it would have to be sooner than originally planned. Once they told us that it was safer to go the IVF route, we didn’t give it a second thought*”.

One partner, Ben, stated: “*It was always going to be in our plans but the diagnosis just bought it forward. There was a window for us to have the opportunity and I wasn’t going to make her wait. I thought that maybe we weren’t ready to have children but are you ever really ready?*”

Whilst another partner, Courtney, spoke about her knowledge of future fertility options and her partner’s lack of understanding around this. She said “*I’ve got all these plans about how we’re going to have kids- I’d like one of mine and one of hers, almost like a surrogate. She knows nothing of that so when I say these things she thinks I’m talking about science fiction.*”

There were some women who despite initially having fertility sparing surgery had recurrence of their tumor and then underwent completion surgery. One participant who had three children had already decided that she would not have any more children. However, amongst other participants, the reactions to this trajectory differed and there was a sense of regret and resignation especially for women who were in new relationships. One participant spoke of her sense of powerlessness and that ‘*it was taken out of her hands*’, explaining, 

Rebecca: “*For the second surgery it was so obvious that I was going to lose the second ovary and would go into early menopause. I think that the whole process from losing my fertility, to going into early menopause- a lot of it was taken out of my hands. I was single, 42 years old, only had 1 ovary and needed IVF- I was aware that there wouldn’t be any more babies.*”

Another highlighted the reality of the inability to have children in the future,

Alice: “*It’s constantly in the back of my mind, there’s something that I can’t provide. Something that actually can’t happen. There’s nothing I can do about that, I can’t be that person.*” 

*Role of partners in decision making and relationship pressures*

Women in this study who were not in a relationship at the time of diagnosis tended to be younger and these women reported less effect on the relationships with their support network. 

Kim: “*The initial process was quite distressing because I am on my own and I don’t have a partner I was relying on friends to come with me. It hasn’t changed my relationships.*”

For women who were in a relationship, the diagnosis of an early stage gynecologic cancer increased pressure on personal relationships. On one hand, some participants reported a strengthening of the relationship: 

Anna: “*It changed the relationship I have with my partner. It was our first year together. It was crazy to go through that together but the support and strength we got from that was nice.*”

However, in several instances, it had contributed to the relationship breakdown. One participant explained, Alice: “*Did this diagnosis put extra pressure on the relationship? Absolutely. Definitely. The sudden loss of fertility affected the relationship with my husband. I had only been married for three months when this all happened and I kicked him out and stopped talking to him for 12 months. I couldn’t deal with him and what was going on. It was very stressful.*”

The participants who underwent IVF reported making that decision in conjunction with their partner and the motivating factor was not wanting to miss out on the opportunity of having a child.

Alice: “*My partner at the time was involved in the decision-making process, we ended up doing fertility treatment and getting the embryos. It was a decision that we made together but I definitely would have driven it.*”

For some couples, the pressure of IVF contributed to the relationship breakdown.

Rebecca: “*Our relationship was pretty broken by then and we thought we would be able to heal it by having a child. The IVF put enormous pressure on us.*”

Furthermore, women who have met a new partner stated that the experiences following diagnosis and treatment have continued to have an impact in their new relationship. There is a change in their sense of self and they have felt the need to discuss fertility options with the new partner.

Alice: *“Definitely this has changed interactions in this relationship. It’s constantly in the back of my mind. There is something that I can’t provide.*” 

May: “*I had a partner at the time but not the same partner as I have now. With the old partner, he was all for the hysterectomy. As circumstances happen, we’re not together and me and my current partner would like to have children.”*

There was also the issue of what to do with embryos that were created with the ex-partner with one participant explaining that she has kept the embryos frozen for eight years. 

Rebecca: *“I couldn’t face letting them go.”*

*Decision for treatment and post-operative regrets*

This theme centred around the sense of not being listened to and perceived lack of control in the decision-making process. Laura spoke of the lack of relevant and timely information to enable her to make treatment decisions and mentioned that she “*didn’t feel supported or listened to*”. She explained the confusion and lack of information which led to her sense of ‘*not being heard*’ in terms of her treatment wishes, 

“*I don’t know if it was because the anesthetist was a woman of about my age who picked up suddenly in my emotions that ‘no, she’s not ready’. I don’t know if it was that one conversation that I had with that one doctor. You have to be thinking ‘hang on, she might not be ready to finish having children’. I was just told, ‘well, that’s the safest thing to do- to get rid of everything’. When I got a call at Christmas and she said that I was an urgent case, hearing that I thought that I was urgent and had to be done.*”

Another participant spoke of the decision being ‘*taken out of my hands*’. 

Rebecca: “*I think that the whole process from losing my fertility, to going into early menopause- a lot of it was taken out of my hands. By that point it was pretty clear that I wouldn’t get to have any more babies.*”

And yet another participant felt that she had little guidance about the role of hysterectomy considering her younger age. 

Kim: “*One doctor did say that I needed to think about whether a hysterectomy would be the right choice for me-I was 32*”.

Patients spoke about continuity of care and there was a contrast in the experience of those women who saw the same clinician and those who saw a different doctor at each clinic appointment. Those who saw a different clinician reported a need for more information. Potentially, they perceived a greater contrast in the information given from different clinicians despite treatment plans being discussed at the multi-disciplinary tumor board meetings. 

Kym: “*I was unprepared for seeing a different doctor every time. It feels impersonal.*”

Laura: “*I know that they are different doctors, I know the information changes.*”

Julie: “*More information between the consults is a definite need, especially when you’re getting the opinions of different doctors. If we could increase continuity and a copy of the follow-up letter that goes to the GP.*”

Continuity of care was also a recurrent theme in the partner interviews and overall partners were very happy with the care that the patients received. 

Courtney: “*She’s seen one of the doctors several times in a row. She really likes him, they have a good rapport. He remembers her and I think that continuity of care is really beneficial for her.*”

Ben: “*Once we knew that things were discussed at tumor board we started to feel more comfortable seeing different doctors, especially when different information is coming from different doctors.*”

Additionally, the option of having a friend or relative attend the clinic appointments was felt to be important. When it came to the time to make decisions about treatment, it was clear that most women made the decision themselves with minimal input from their partner or support network but they appreciated the opportunity to rediscuss what was said after the appointment. 

Sophie: “*I made this decision on my own and I think that I would still make the decision for myself.*”

Some participants expressed regret about their decisions to have a hysterectomy.

Alice: “*I would definitely say that the fertility side of my diagnosis is my biggest regret. I don’t feel like I should have had the hysterectomy. I don’t feel that it should have happened at all. Overarching feeling of regret.*”

May: “*To think about it now, it makes me feel pretty ripped off. In hindsight I wouldn’t have had it done.*”

*Patient rationale for partners not interviewing*

Partner recruitment remained low throughout the study. Participants spoke of their autonomy in treatment decisions and of their partner being supportive of their decisions. Two participants spoke of their partners prioritising their needs first. One partner reportedly stated that he felt he had ‘*little to add*’. 

Anna: “*I showed him your letter and we had a little chat. The main thing that he wanted was for me to get better. He was there for me for that. He saw it as my choice. It’s not a traditional relationship, it was always my decision and it was still in the early days of our relationship. He just wanted me to be better, to recover and make sure that everything was fine. He didn’t think that he could bring anything to the survey.*”

Hannah: “*At the time of making the decision, I talked with him, I told him what I had, he was just supportive. He said to do what I needed to do*”.

Laura: “*He just would not. Even me saying that I’m going to do it, he was like “what are you doing that for?*”*. He kinda just left it up to me. It was all my decisions, I wasn’t even telling him that I was going for my appointments.*”

**Table 1:** Patient Age, Fertility Data and Gynecological Cancer Type

Patient Age	Responders (n=14)	Non-responders (n=32)
	33 years (St dev 6.9)	30 years (St Dev 3.6)
Diagnosis		
Cervical tumors 6		Cervical tumours 22
Uterine corpus tumors 3		Uterine corpus tumors 6
Ovarian tumors 5		Ovarian tumors 4
Parity pre-diagnosis:		
Para 0 7		
Para 1 3		
Para 2 2		
Para 3 3		
Events post diagnosis:		
4 attempted IVF		
1 child conceived post IVF		
4 separated from partner		
5 remained in same relationship		
5 not in a relationship		
2 re-partnered		
3 underwent completion surgery during the study time frame		

**Table 2:** Key Themes Derived from Interview Discussion with Patients and Their Partners

Key Findings for Participants
• Receiving a cancer diagnosis is an emotional timepoint in a young woman’s life.
• Women wanted to bring support people to their appointments.
• Partners were reported as supportive. Women expressed that the final decision for treatment was their decision; there was little influence from partners.
• Access to additional services such as psychologists and social work was appropriate. The option to discuss factors in the decision-making process with a counselor or psychologist was beneficial.
• Women who had undergone completion surgery expressed a degree of regret and would advise other women to “only do this if you’re ready”.
Key findings for partners
• Partners wanted to be supportive and would like more information to be available before and after the clinic appointments.
• Partners felt respected by healthcare workers.
• Continuity of care was appreciated by both the partner and the patient. At times the partners would have liked the treating doctors to be more up to date in the knowledge of the patient journey as well as the next management step.
• Partners were pleased with the care that their loved-one received.

## Discussion

Few studies have investigated how women perceive the information given to them at the time of diagnosis of an early stage gynecologic cancer. In particular, how much information is sought regarding fertility options such as IVF or the ability for spontaneous conception. In the current study several participants expressed a sense of uncertainty, the loss of future self and fear of the worst-case scenario. Half of participants reported that the diagnosis put significant pressure on close relationships resulting in relationship breakdown. This finding is in keeping with other studies around the pressure that illness brings to bear on relationships with significant others (Manne and Badr, 2008; Rosen et al., 2009; Reh et al., 2011).

Patients requested more information about the oncology service in the form of an introductory letter, more information between consults such as a summary letter, improved continuity of care (same patient care clinician or lead consultant) and greater consistency of information given. Women drew comfort in the knowledge that there was a multidisciplinary team discussing their case and treatment options. Importantly, women wanted the ability to make the decision about their care without external pressures. 

There was a high rate of regret in those women who did not feel in control of the decision- making process. Patients who actively participate in their care are more satisfied (Roberts et al., 1997; Wallberg et al., 2000; Keating et al., 2010). Several women reiterated the importance of feeling that they are able to ask questions, as well as medical staff not taking the first answer as the patient’s final answer: when asking women if they have finished their family give them time to reflect, “*if someone says ‘No.’, ask ‘Are you sure?’”*. 

Limitations of the study include a low response rate and a small sample that consisted primarily of patients of Australian or Western European origin. Thus, caution should be exercised when considering the generalizability of findings to other patient populations. An additional limitation was that only two partners were recruited. Further, there was a high number of non-responders despite utilising an opt-out method of recruitment, which may have introduced bias. 

This study aimed to explore attitudes to conception in young women who undertook fertility sparing surgery for the treatment of early stage gynecologic cancer. Our findings are consistent with previous reports in which up to 59% of women with cancer rated having children as the most important issue in their life (Lagana et al.; Reh et al., 2011; Zapardiel et al., 2016). Participants in the current study felt that counseling and their clinical experience could have been improved through better communication, invitations to include friends or family, improved continuity of care, and clinician understanding of individual patient factors. There was a high rate of regret associated with completion surgery. We have highlighted the complexity of decision making these young women face at a confronting and emotional time of their lives. Future research should include studies of interventions aimed at improving clinician communication and reducing regret in this patient group.

In conclusion, in this study of young women with gynecologic cancers undergoing fertility-sparing treatment four main themes were identified: (i) Emotions at diagnosis and perception of information given; (ii) Discussions of fertility and factors affecting childbearing; (iii) Role of partners in decision making and relationship pressures; (iv) Decision for treatment and postoperative regrets. Regret and relationship breakdown were commonly reported. Patients with gynecologic cancers undergoing fertility-sparing treatment need appropriate support including inviting their partners to attend clinic appointments. Women may need several appointments prior to their treatment.

*Relevance to Clinical Practice*

This was a qualitative study that aimed to explore attitudes to conception in partners and young women with gynecologic cancers treated by fertility sparing surgery. In higher income countries there is a trend to late childbearing, resultantly, fertility preservation is becoming more important for all women. In higher income countries there is a trend to late childbearing and fertility preservation is becoming more important for many women. For women of reproductive age who are diagnosed with early stage and potentially curable gynecologic disease, neither the patient experience or the decision-making process leading to fertility preservation has been widely explored. Currently a majority of the data relating to what patients experience throughout their diagnosis, management and follow-up is derived from breast cancer research with limited studies from gynecology. This study highlights that illness increases pressure on close personal relationships through a loss of self-identity and increased fear for the future. Patients reported increased stressors related to decisions surrounding their fertility, perceived external pressures and a loss of control. Participants in the current study felt that their clinical experience could have been improved through better communication, invitations to include friends or family, improved continuity of care, and clinician understanding of individual patient factors. There was a high rate of regret associated with completion surgery. We have highlighted the complexity of decision making these young women face at a confronting and emotional time of their lives.
